# Phosphorylation of p65 Is Required for Zinc Oxide Nanoparticle–Induced Interleukin 8 Expression in Human Bronchial Epithelial Cells

**DOI:** 10.1289/ehp.0901635

**Published:** 2010-03-01

**Authors:** Weidong Wu, James M. Samet, David B. Peden, Philip A. Bromberg

**Affiliations:** 1 Center for Environmental Medicine, Asthma, and Lung Biology, University of North Carolina, Chapel Hill, North Carolina, USA; 2 Human Studies Division, National Health and Environmental Effects Research Laboratory, U.S. Environmental Protection Agency, Research Triangle Park, North Carolina, USA

**Keywords:** bronchial epithelial cells, IL-8, interleukin-8, NFκB, p65, zinc oxide

## Abstract

**Background:**

Exposure to zinc oxide (ZnO) in environmental and occupational settings causes acute pulmonary responses through the induction of proinflammatory mediators such as interleukin-8 (IL-8).

**Objective:**

We investigated the effect of ZnO nanoparticles on *IL-8* expression and the underlying mechanisms in human bronchial epithelial cells.

**Methods:**

We determined *IL-8* mRNA and protein expression in primary human bronchial epithelial cells and the BEAS-2B human bronchial epithelial cell line using reverse-transcriptase polymerase chain reaction and the enzyme-linked immunosorbent assay, respectively. Transcriptional activity of *IL-8* promoter and nuclear factor kappa B (NFκB) in ZnO-treated BEAS-2B cells was measured using transient gene transfection of the luciferase reporter construct with or without *p65* constructs. Phosphorylation and degradation of IκBα, an inhibitor of NF-κB, and phosphorylation of p65 were detected using immunoblotting. Binding of p65 to the *IL-8* promoter was examined using the chromatin immunoprecipitation assay.

**Results:**

ZnO exposure (2–8 μg/mL) increased *IL-8* mRNA and protein expression. Inhibition of transcription with actinomycin D blocked ZnO-induced *IL-8* expression, which was consistent with the observation that ZnO exposure increased *IL-8* promoter reporter activity. Further study demonstrated that the κB-binding site in the *IL-8* promoter was required for ZnO-induced *IL-8* transcriptional activation. ZnO stimulation modestly elevated IκBα phosphorylation and degradation. Moreover, ZnO exposure also increased the binding of p65 to the *IL-8* promoter and p65 phosphorylation at serines 276 and 536. Overexpression of *p65* constructs mutated at serines 276 or 536 significantly reduced ZnO-induced increase in *IL-8* promoter reporter activity.

**Conclusion:**

p65 phosphorylation and IκBα phosphorylation and degradation are the primary mechanisms involved in ZnO nanoparticle-induced *IL-8* expression in human bronchial epithelial cells.

Inhalation of zinc oxide (ZnO) particles can provoke a number of clinical responses of which the best known is metal fume fever (Gordon et al. 1992; Kuschner et al. 1995). This is accompanied by changes in composition of bronchoalveolar lavage fluid, including early increase in tumor necrosis factor α (TNFα) followed by interleukin (IL)-8 and IL-6, and in numbers of polymorphonuclear leukocytes (Blanc et al. 1993; Kuschner et al. 1995, 1997).

ZnO particles in ambient air arise from incinerator emission and from wear and tear of vehicle tires (Adachi and Tainosho 2004; Bennett and Knapp 1982; Councell et al. 2004; Horner 1996; Lough et al. 2005). Previous studies have demonstrated that exposure to Zn-laden (Zn in its salt and oxidized forms) ambient particles contribute to the increase in bronchitis and asthma morbidity and in lung toxicity (Adamson et al. 2000, 2003; Hirshon et al. 2008; Pope 1989). It is noteworthy that engineered ZnO nanoparticles (< 100 nm in diameter) are currently being produced in high tonnage (Xia et al. 2008). Exposure to these nanoparticles may occur in occupational, consumer, and environmental settings (Stone et al. 2007). Inhaled nanoparticles can deposit along the entire respiratory tract, including airways and alveolar regions (Yang et al. 2008). Recent *in vitro* studies have revealed that ZnO nanoparticles had a stronger effect on induction of cell damage to human alveolar epithelial cells and on IL-8 production from human bronchial epithelial cells and aortic endothelial cells compared with other metal oxide nanoparticles (Gojova et al. 2007; Park et al. 2007; Xia et al. 2008).

IL-8, a member of the CXC chemokine family, is an important activator and chemoattractant for polymorphonuclear leukocytes and has been implicated in a variety of inflammatory diseases (Strieter 2002). IL-8 protein is secreted at low levels from nonstimulated cells, but its production is rapidly induced by a wide range of stimuli encompassing proinflammatory cytokines (Kasahara et al. 1991), bacterial or viral products (Hobbie et al. 1997; Johnston et al. 1998), and cellular stressors (Fritz et al. 2005; Hirota et al. 2008; Kafoury and Kelley 2005; Sonoda et al. 1997). Expression of the *IL-8* gene is regulated primarily at the level of transcription, although contributions by posttranscriptional mechanisms such as mRNA stabilization have also been demonstrated (Holtmann et al. 1999, 2001; Roebuck 1999; Winzen et al. 1999). The *IL-8* gene is located on human chromosome 4, q12–21, and consists of four exons and three introns. Its 5′-flanking region contains the usual CCAAT and TATA boxlike structures and a number of potential binding sites for several inducible transcription factors including nuclear factor kappa B (NFκB), activator protein-1 (AP-1), and CAAT/enhancer-binding protein (C/EBP) (Luster 1998; Roebuck 1999; Wu et al. 1997). Regulation of *IL-8* gene transcriptional activation is stimulus and cell-type specific (Brasier et al. 1998; Kasahara et al. 1991; Medin and Rothman 2006; Roebuck et al. 1999; Strieter 2002), which requires a functional NFκB element in addition to either an AP-1 or a C/EBP (NF-IL-6) element under some conditions of transcriptional induction (Strieter 2002). Unlike the NFκB site, the AP-1 and C/EBP sites are not essential for induction but are required for maximal gene expression of the *IL-8* gene (Hoffmann et al. 2002). Although ZnO induces IL-8 expression in bronchial epithelial cells and IL-8 plays a critical role in the pathogenesis of pulmonary disorders (Blanc et al. 1993; Kuschner et al. 1997, 1998; Standiford et al. 1993), the mechanisms underlying ZnO-induced *IL-8* expression have not been well characterized. In this study, we investigated the regulatory mechanisms underlying ZnO-induced *IL-8* expression in human bronchial epithelial cells.

## Materials and Methods

### Materials and reagents

We purchased ZnO (99% purity, 24–70 nm in diameter) from Alfa Aesar (Ward Hill, MA); Triton X-100 and polyacrylamide from Sigma Chemical Co. (St. Louis, MO); and SDS-PAGE (sodium dodecyl sulfate polyacrylamide gel electrophoresis) supplies, such as molecular mass standards and buffers, from Bio-Rad (Richmond, CA). We obtained anti-human p65 polyclonal antibody from Cayman Chemical (Ann Arbor, MI); phospho-specific rabbit antibodies against human NFκB p65 [serine 276 (Ser276), serine 536 (Ser536)] and human IκBα [serine 32 (Ser32)] from Cell Signaling Technology (Beverly, MA); β-actin antibody from USBiological (Swampscott, MA); and IκBα antibody and horseradish peroxidase (HRP)-conjugated goat anti-rabbit antibody from Santa Cruz Biotechnology (Santa Cruz, CA). Actinomycin D (Act D) was purchased from EMD Biosciences, Inc. (San Diego, CA); the IL-8 ELISA (enzyme-linked immunosorbent assay) kit and the recombinant TNFα were purchased from eBioscience (San Diego, CA); the FuGENE 6 transfection reagent was obtained from Roche Diagnostics Corporation (Indianapolis, IN); and chemiluminescence reagents were obtained from Pierce Biotechnology (Rockford, IL).

### Cell culture and exposure

#### Primary human bronchial epithelial cells

According to a protocol approved by the University of North Carolina Institutional Review Board, cells were obtained by cytologic brushing during bronchoscopy from healthy nonsmoking adult volunteers who had given informed consent; cells were frozen in liquid nitrogen until use. After thawing, the human bronchial epithelial cells were expanded to passage 2 in bronchial epithelial growth medium (Cambrex Bioscience Walkersville, Inc., Walkersville, MD), then plated on collagen-coated filter supports with a 0.4-μm pore size (Trans-CLR; Costar, Cambridge, MA) to undergo air liquid interface (ALI) culture in a 1:1 mixture of bronchial epithelial cell basic medium and Dulbecco’s modified Eagle’s medium-H with SingleQuot supplements (Cambrex), bovine pituitary extract (13 mg/mL), bovine serum albumin (1.5 μg/mL), and nystatin (20 units). Upon confluency, *all-trans* retinoic acid was added to the medium, and ALI culture conditions (removal of the apical medium) were created to promote differentiation. ZnO nanoparticles were suspended in molecular-grade water. Because ZnO nanoparticles were poorly dissolved in water, the ZnO suspension was sonicated before being added to the apical surface of the ALI culture for stimulation.

#### BEAS-2B cell line

The BEAS-2B cell line was derived by transforming human bronchial cells with an adenovirus 12-simian virus 40 construct (Reddel et al. 1988). We obtained BEAS-2B cells from the American Type Culture Collection (ATCC, Manassas, VA). BEAS-2B cells (passages 70–80) were grown on tissue culture–treated Costar plates in keratinocyte basal medium supplemented with 30 μg/mL bovine pituitary extract, 5 ng/mL human epidermal growth factor, 500 ng/mL hydrocortisone, 0.1 mM ethanolamine, 0.1 mM phosphoethanolamine, and 5 ng/mL insulin. A suspension of ZnO was added to the surface of confluent BEAS-2B cells for stimulation. The doses of ZnO nanoparticles used in this study ranged from 2 to 8 μg/mL.

### Real-time reverse transcriptase/polymerase chain reaction (RT-PCR)

Bronchial epithelial cells grown to confluence were exposed to ZnO. Cells were washed with ice-cold phosphate-buffered saline (PBS) and then lysed with TRIZOL reagent (Invitrogen Corporation, Carlsbad, CA). Total RNA (100 ng), 0.5 mM nucleoside triphosphate (Pharmacia, Piscataway, NJ), 5 μM random hexaoligonucleotide primers (Pharmacia), 10 U/μL RNase inhibitor (Promega, San Luis Obispo, CA), and 10 U/μL Moloney murine leukemia virus RT (GIBCO-BRL Life Technologies, Gaithersburg, MD) were incubated in a 40°C water bath for 1 hr in 50 μL 1× PCR buffer to synthesize first-strand cDNAs. The reverse transcription was inactivated by heating at 92°C for 5 min. Quantitative PCR of *IL-8* and glyceraldehyde-3-phosphate dehydrogenase (*GAPDH*) specimen cDNA and standard cDNA was performed in a 50-μL final volume mixture containing TaqMan master mix (PerkinElmer, Foster City, CA), 1.25 μM probe, 3 μM forward primer, and 3 μM reverse primer. The probe annealed to the template between the two primers. This probe contained both a fluorescence reporter dye at the 5′ end (6-carboxyfluorescein: emission λ_max_ = 518 nm) and a quencher dye at the 3′ end (6-carboxytetramethyl rhodamine: emission λ_max_ = 582 nm). During polymerization, the probe was degraded by the 5′–3′ exonuclease activity of the Taq DNA polymerase, and the fluorescence was detected by a laser in the sequence detector (TaqMan ABI Prism 7700 Sequence Detector System; PerkinElmer). Thermal cycler parameters included 2 min at 50°C, 10 min at 95°C, and 40 cycles of denaturation at 95°C for 15 sec and annealing/extension at 60°C for 1 min. Relative amounts of *IL-8* and *GAPDH* mRNA were based on standard curves prepared by serial dilution of cDNA from human BEAS-2B cells. The oligonucleotide primers and probes were purchased from Applied Biosystems (Foster City, CA).

### ELISA

We assayed IL-8 protein in the cell culture supernatant using with a human IL-8 ELISA kit according to the manufacturer’s instructions.

### Immunoblotting

BEAS-2B cells were treated with ZnO suspension, washed twice with ice-cold PBS, then lysed in RIPA buffer [1× PBS, 1% nonidet P-40, 0.5% sodium deoxycholate, 0.1% SDS, and protease inhibitors (20 μg/mL leupeptin, 20 μg/mL aprotinin, 0.5 mM phenylmethylsulfonyl fluoride, 200 μM sodium orthovanadate, and 20 mM sodium fluoride)]. Supernatants of cell lysates were subjected to SDS-PAGE. Proteins were transferred onto nitrocellulose membrane. The membrane was blocked with 5% nonfat milk, washed briefly, and incubated with primary antibody at 4°C overnight, followed by incubation with corresponding HRP-conjugated secondary antibody for 1 hr at room temperature. Immunoblot images were detected using chemiluminescence reagents and the Gene Gynome Imaging System (Syngene, Frederick, MD).

### Chromatin immunoprecipitation (ChIP) assay

We conducted the ChIP assay using a ChIP kit (Upstate, Lake Placid, NY). Briefly, BEAS-2B cells growing in 100-mm dishes were treated with ZnO for 2 hr before being subjected to cross-linking with 1% formaldehyde at 37°C for 10 min. After washing with PBS, the cells were resuspended in 300 μL lysis buffer [50 mM Tris-HCl (pH 8.1), 10 mM EDTA, 1% SDS, protease inhibitor cocktail]. DNA was sheared to 200–1,000 base pair small fragments by sonication. The supernatant was recovered, diluted, and precleared using salmon sperm DNA/protein A agarose. The recovered supernatant was incubated with anti-p65 antibody or an isotype control IgG for 2 hr in the presence of salmon sperm DNA and protein G-Sepharose beads. The beads were washed with low-salt, high-salt, and lithium chloride buffers. The immunoprecipitated DNA was retrieved from the beads with 1% SDS and 0.1 mM NaHCO_3_ solution at 65°C for 4 hr, then purified with a QIAquick spin column (Qiagen, Valencia, CA). The PCR was conducted on the extracted DNA using *IL-8* promoter-specific primers at 95°C for 2 min, followed by 35 cycles of 95°C for 30 sec, 55°C for 30 sec, and 72°C for 30 sec. The PCR products were separated on a 1.4% agarose gel and stained with ethidium bromide.

### Transient gene transfection

BEAS-2B cells grown to 40–50% confluence were transfected with the specific constructs for 24 hr using FuGENE 6 transfection reagent according to manufacturer’s instructions. These constructs included luciferase-conjugated *IL-8* promoter (p1.5*IL-8*-luc), κB binding-site–mutated *IL-8* promoter (p1.5*IL-8*-κB-luc), the five tandem repeat of NFκB response element (pNFκB-luc), and Flag-*p65*, Flag-*p65* S276A, and FLAG-*p65* S536A (Kim et al. 2007). The pSV-β-galactosidase construct was co-transfected with the individual construct depicted previously as an internal control (Kim et al. 2007). After transfection, the cells were incubated with keratinocyte basal medium overnight and then treated with ZnO before being lysed with lysis buffer. We detected luciferase and β-galactosidase activities using the Dual-Light chemiluminescent reporter gene assay system (Tropix, Bedford, MA) and an AutoLumat LB953 luminometer (Berthold Analytical Instruments, Nashua, NH). Luciferase activity was estimated as luciferase count/β-galactosidase count (luc/gal).

### Statistical analysis

Data are presented as mean ± SE. Data were evaluated using nonparametric paired *t*-tests with the overall α level set at 0.05. One-way analysis of variance was used to analyze the time- and dose-dependent trends of *IL-8* mRNA and protein expression.

## Results

### ZnO exposure increases IL-8 expression in human bronchial epithelial cells

**Figure 1:**
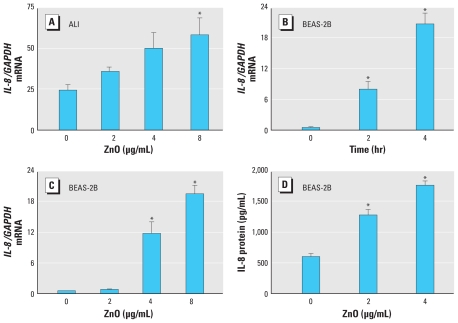
ZnO exposure increases *IL-8* mRNA and protein expression in human bronchial epithelial cells. (*A*) *IL-8/GAPDH* mRNA level in ALI-cultured primary human bronchial epithelial cells treated with 2–8 μg/mL ZnO, (*B*) confluent BEAS-2B cells treated with 8 μg/mL ZnO for 2 hr or 4 hr, and (*C*) confluent BEAS-2B cells treated with 2, 4, or 8 μg/mL ZnO for 4 hr. *IL-8* mRNA levels were determined by RT-PCR. (*D*) IL-8 protein in BEAS-2B cells treated with 4 or 8 μg/mL ZnO for 6 hr; assayed protein content was determined using ELISA. Data shown are representative of three separate experiments. *p < 0.05 compared with 0 μg/mL ZnO.

To examine the effect of ZnO nanoparticles on *IL-8* expression in human bronchial epithelial cells, we used ALI-cultured primary human bronchial epithelial cells and BEAS-2B cells. As shown in Figure 1A, exposure of ALI-cultured primary human bronchial epithelial cells to 8 μg/mL ZnO for 4 hr induced a significant increase in *IL-8* mRNA levels. In BEAS-2B cells, ZnO stimulation (8 μg/mL) induced a time-dependent increase in *IL-8* mRNA expression (*F* = 47.24; *p* < 0.01). Exposure of BEAS-2B cells to ZnO for 4 hr caused a dose-dependent increase in *IL-8* mRNA expression (Figure 1C; *F* = 41.83, *p* < 0.01). In addition, ZnO exposure resulted in a dose-dependent increase in IL-8 protein release from BEAS-2B cells after a 6-hr exposure (Figure 1D; *F* = 96.14, *p* < 0.01). These results indicate that ZnO exposure up-regulates *IL-8* expression at both mRNA and protein levels in human bronchial epithelial cells.

### Transcriptional activation is involved in ZnO-induced IL-8 expression

**Figure 2:**
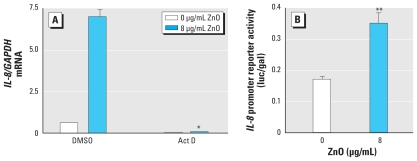
Transcriptional regulation is involved in ZnO-induced *IL-8* expression. (*A*) *IL-8/GAPDH* mRNA level in confluent BEAS-2B cells pretreated with 10 μg/mL Act D for 30 min, then stimulated with 8 μg/mL ZnO for 2 hr. Dimethyl sulfoxide served as a vehicle control for Act D. *IL-8* mRNA levels were determined by RT-PCR. (*B*) *IL-8* promoter reporter activity in BEAS-2B cells grown to 40–50% confluence and transfected with p1.5*IL-8-*luc and pSV-β-galactosidase constructs using FuGENE 6 transfection reagent. Twenty-four hours after transfection, cultures were incubated with keratinocyte basal medium overnight; the cells were then treated with 8 μg/mL ZnO for 6 hr before being lysed. Luciferase activity was estimated as luciferase count/β-galactosidase count (luc/gal). Data shown are representative of three separate experiments. *p < 0.05 compared with DMSO plus ZnO. **p < 0.05 compared with 0 μg/mL ZnO.

To examine the involvement of transcriptional regulation in ZnO-induced elevation of *IL-8* mRNA, we pretreated BEAS-2B cells with 10 μg/mL Act D, a potent inhibitor of RNA polymerase, before treatment with ZnO. Pretreatment with Act D for 30 min ablated ZnO-induced *IL-8* mRNA (Figure 2A), suggesting that transcriptional regulation was required for *IL-8* expression in ZnO-exposed cells. To confirm this observation, we measured the *IL-8* promoter activity through transient gene transfection of a luciferase-conjugated *IL-8* promoter construct. As predicted by the Act D results, ZnO exposure (8 μg/mL) significantly increased *IL-8* promoter reporter activity (Figure 2B). These data indicate that ZnO-induced *IL-8* gene expression in human bronchial epithelial cells occurs through a transcriptional mechanism.

### ZnO exposure induces NFκB activation

**Figure 3:**
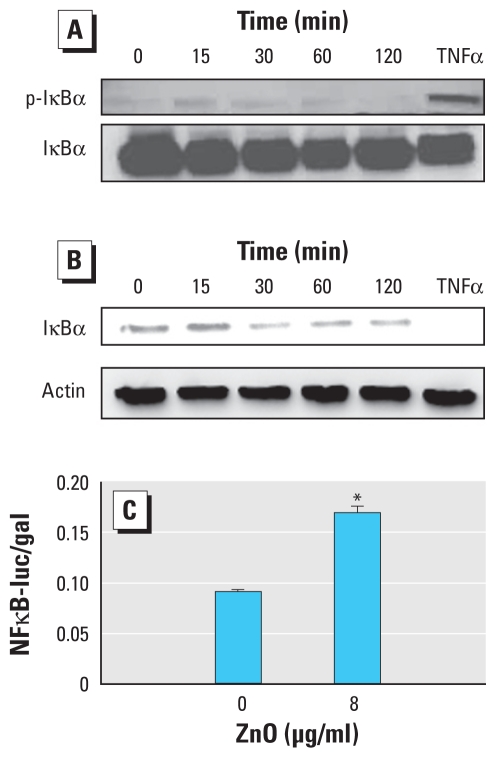
ZnO exposure induces NFκB activation in BEAS-2B cells. (*A*) Phosphorylation of IκBα in cells pretreated with 20 μM MG132 for 30 min (to prevent IκBα degradation) before further stimulation with 8 μg/mL ZnO for 15, 30, 60, or 120 min, or 100 ng/mL TNFα for 30 min; cell lysates were separated by SDS-PAGE and immunoblotted with anti-phospho-IκBα (Ser32/36) and anti-IκBα antibodies. (*B*) IκBα degradation in cells treated with 8 μg/mL ZnO for 15, 30, 60, or 120 min, or 100 ng/mL TNFα for 30 min; cell lysates were separated by SDS-PAGE and immunoblotted with anti-IκBα and anti-actin antibodies. (*C*) NFκB reporter activity, estimated as luciferase count/β-galactosidase count, in cells grown to 40–50% confluence, transfected with pNFκB-luc and p-*SV-*β-galactosidase constructs, and treated with 8 μg/mL ZnO for 6 hr. Data shown are representative of three separate experiments. *p < 0.05 compared with 0 μg/mL ZnO.

Activation of the transcription factor NFκB is required for *IL-8* gene transcription activation in many cell types (Villarete and Remick 1996). To examine whether ZnO stimulation increased NFκB activity, we determined phosphorylation and degradation of the NFκB inhibitory protein κBα (IκBα), an event indicative of the canonical NFκB-activating pathway (Ghosh and Karin 2002; Karin and Ben-Neriah 2000). In BEAS-2B cells exposed to 8 μg/mL ZnO for 15, 30, 60, or 120 min, we measured phosphorylation of IκBα on Ser32. As shown in Figure 3A, exposure to ZnO (after pretreatment with the proteasome inhibitor MG132) induced a modest phosphorylation of IκBα, which peaked at 15-min exposure and declined thereafter. As expected, TNFα (100 ng/mL), the positive IκBα phosphorylation inducer, increased IκBα phosphorylation at 30 min exposure. In a manner consistent with this observation, ZnO stimulation in the absence of MG132 caused IκBα degradation at 30 min exposure to ZnO (Figure 3B). These data indicated that ZnO exposure can induce modest canonical NFκB activation in BEAS-2B cells. To further confirm ZnO-induced NFκB activation, BEAS-2B cells were transiently transfected with pNFκB-luc and p-SV-β-galactosidase constructs prior to ZnO treatment. As shown in Figure 3C, ZnO exposure (8 μg/mL) increased NFκB reporter activity at 6 hr of exposure, demonstrating that ZnO treatment increases NFκB-dependent transcriptional activity.

### NFκB is required for ZnO-induced IL-8 expression

**Figure 4:**
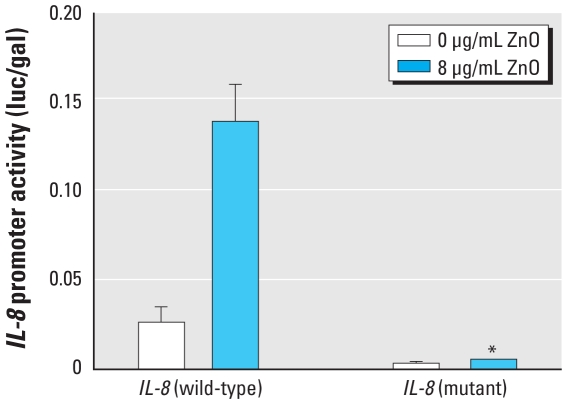
NFκB is required for ZnO-induced *IL-8* gene transcription, as shown by luciferase activity. BEAS-2B cells grown to 40–50% confluence were transfected with p1.5*IL-8*-luc (*IL-8* wild-type) or p1.5*IL-8*-κB-luc (*IL-8* mutant), as described in “Materials and Methods,” prior to treatment with 8 μg/mL ZnO for 6 hr. Data shown are representative of three separate experiments. *p < 0.05 compared with ZnO treatment in the *IL-8* wild-type group.

To further determine whether NFκB was involved in ZnO-induced *IL-8* gene transcription, we transfected BEAS-2B cells with luciferase-conjugated *IL-8* promoter (p1.5*IL-8*-luc) and κB binding site–mutated *IL-8* promoter (p1.5*IL-8*-κB-luc) constructs, respectively, prior to treatment with 8 μg/mL ZnO for 6 hr. As shown in Figure 4, the luciferase reporter activity induced by ZnO stimulation was significantly reduced in the cells expressing κB binding site–mutated *IL-8* promoter compared with that in the cells expressing the intact (wild-type) *IL-8* promoter, implying that NFκB is required for ZnO-induced *IL-8* gene transcription.

### Phosphorylation of p65 NFκB mediates ZnO-induced IL-8 mRNA expression

**Figure 5:**
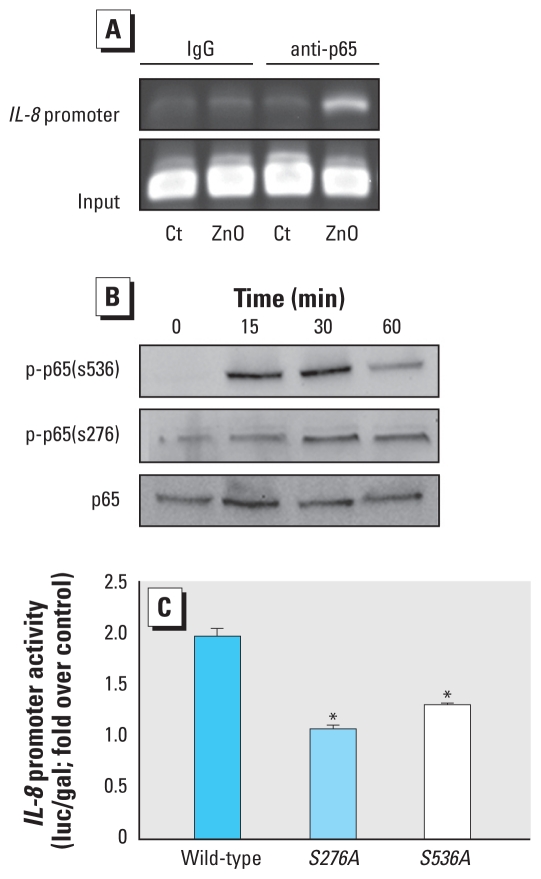
Phosphorylation of p65 NFκB mediates ZnO-induced *IL-8* gene transcription in BEAS-2B cells. (*A*) Binding of the *IL-8* gene promoter in cells treated with 8 μg/mL ZnO for 2 hr, as detected by the ChIP assay using anti-p65 antibody. Precipitates from the antibody against IgG served as a negative control, and 2% of the diluted DNA was used as loading control. The target sequence for PCR was located around the *IL-8* gene promoter. (*B*) Phosphorylation of p65 NFκB in cells treated with 8 μg/mL ZnO for 15–60 min and analyzed by SDS-PAGE and immunoblotting with anti-phospho-p65/RelA (Ser536), anti-phospho-p65/RelA (Ser276), or anti-p65/RelA antibodies. (*C*) *IL-8* promoter reporter activity, estimated as luciferase count/β-galactosidase count, in cells grown to 40–50% confluence and co-transfected with wild-type *p65* construct or a mutated version, as described in “Materials and Methods,” prior to treatment with 8 μg/mL ZnO for 6 hr. Data shown are representative of three separate experiments. *p < 0.05 compared with the *p65* wild-type group.

NFκB exerts its regulatory function through binding specific DNA sequences as homo- or heterodimers composed of members of the Rel/NFκB family (Hayden and Ghosh 2004). The most ubiquitous NFκB complex is the heterodimer p50/p65(RelA). We investigated whether p65 NFκB could bind to the *IL-8* gene promoter in ZnO-treated BEAS-2B cells incubated with 8 μg/mL ZnO for 2 hr. Cell lysates were then subjected to the ChIP assay using anti-p65 and isotype control antibodies. As shown in Figure 5A, ZnO stimulation resulted in a marked increase in the binding of p65 NFκB to the *IL-8* gene promoter.

Phosphorylation of specific serine residues of the p65 NFκB subunit has been shown to be important for its transcriptional activity (Okazaki et al. 2003). Therefore, we used phospho-specific antibodies to measure the phosphorylation of p65 NFκB at ser276 and ser536 in BEAS-2B cells exposed to ZnO. We observed that ZnO treatment induced a rapid increase in phosphorylation of p65 at both serine residues (Figure 5B). Phosphorylation of p65 (Ser536) in ZnO-treated cells occurred as early as 15 min exposure and was decreased at 60 min. In contrast, phosphorylation levels of p65 (Ser276) went up more slowly but were still above the control level at 60 min. These data indicated that ZnO exposure increased p65 phosphorylation in human bronchial epithelial cells.

To determine the functional importance of p65 phosphorylation in ZnO-induced *IL-8* gene transcription, we co-transfected BEAS-2B cells with p1.5*IL-8*-luc and either a wild-type *p65* construct or a mutated version in which either Ser276 or Ser536 in *p65* was mutated. As expected, ZnO exposure induced increased *IL-8* promoter reporter activity in cells expressing wild-type *p65* (Figure 5C). In comparison with the cells expressing wild-type *p65*, the cells that expressed mutated *p65* showed a significant reduction in *IL-8* promoter reporter activity after ZnO treatment. These data strongly suggest that phosphorylation of p65 is required for ZnO-induced *IL-8* gene transcription.

## Discussion and Conclusion

Cellular responses to environmental stimuli require rapid and accurate transmission of signals from cell-surface receptors to the nucleus (Karin and Hunter 1995). These signaling pathways rely on protein phosphorylation and, ultimately, lead to the activation of specific transcription factors that induce the expression of appropriate target genes. In the present study using human bronchial epithelial cells, we have demonstrated that exposure to the ZnO nanoparticles induces *IL-8* gene expression by activating NFκB through a bimodal mechanism that involves p65 NFκB phosphorylation as well as IκBα phosphorylation and degradation.

Increased expression of IL-8 protein is largely dependent on transcriptional activation of the *IL-8* gene (Wickremasinghe et al. 1999). This is corroborated by the results of the present study, which show that pretreatment of BEAS-2B cells with the transcriptional inhibitor Act D abrogated ZnO-induced *IL-8* mRNA expression as well as the activation of *IL-8* promoter activity in ZnO-treated cells. The NFκB family of transcription factors is essential for inflammation, immunity, and cell proliferation. Five members of the NFκB family have been identified: NFκB1 (p50/p105), NFκB2 (p52/p100), RelA (p65), RelB, and c-Rel. They share a highly conserved Rel homology domain at the N-terminal end that is responsible for specific DNA binding, dimerization, and interaction with IκB. In addition, some Rel proteins such as p65 (RelA) contain one or two C-terminal transactivating domain. In mammals, the NFκB transcription factor consists of two subunits of either homo- or heterodimers of RelA/p65, c-Rel, and p50. The p50/RelA(p65) heterodimer is the major Rel/NFκB complex in most cell types (Gilmore 1999). In resting cells, NFκB complexes are sequestered in an inactive form in the cytoplasm of the cells through its association with an inhibitory protein belonging to the IκB family, IκBα being the prototype. Upon cell stimulation, IκBα is phosphorylated by one of a number of IκB kinases, ubiquitinylated, and degraded, thereby allowing the NFκB complex to translocate into the nucleus and regulate the expression of its target genes, such as those coding for cytokines, adhesion molecules, and chemokines that have a crucial role in both immune and inflammatory responses (Hayden and Ghosh 2004; Karin and Ben-Neriah 2000; Rossi and Zlotnik 2000). Our data show that ZnO exposure induces rapid phosphorylation and degradation of IκBα, consistent with an increase in NFκB transcriptional activity and ensuing *IL-8* gene transcription. However, the modest degree of phosphorylation and degradation of IκBα induced by ZnO stimulation implied that other events might also participate in ZnO-induced NFκB transcriptional activation.

Increasing evidence from biochemical and genetic experiments strongly suggests that optimal induction of NFκB target genes also requires posttranslational modifications of NFκB p65 (Perkins 2006; Schmitz et al. 2004). For example, the acetylation of p65 has been proposed to facilitate the retention of the NFκB complex in the nucleus (Ashburner et al. 2001; Chen et al. 2001). The phosphorylation of p65 can result in a conformational change that increases its DNA binding activity and ability to recruit histone acetyltransferases such as cAMP response element-binding (CREB)-binding protein and p300 and to displace p50–histone deacetylase-1 complexes from DNA, leading to increased transcriptional activity (Ashburner et al. 2001; Chen et al. 2001). The NFκB p65 can be phosphorylated at multiple sites either in the N-terminal Rel homology domain or C-terminal transactivating domain (Viatour et al. 2005). The best-characterized phosphorylated residues on p65 are Ser276 and Ser536. In the present study, we observed that ZnO exposure increased the binding of p65 to the *IL-8* gene promoter and also increased the phosphorylation of p65 at Ser276 and Ser536 in BEAS-2B cells. Previous studies have shown that p65 can be phosphorylated by a variety of cytoplasmic and nuclear kinases in a stimulus- and cell type-specific manner (Jamaluddin et al. 2007; Schmitz et al. 2004; Viatour et al. 2005; Zhong et al. 2002). Further study will be required to elucidate the mechanisms responsible for ZnO-induced phosphorylation of p65.

The role of particle dissolution in ZnO-induced toxic effects has been investigated; however, the results differ with particle size and cell types. A study using human aortic endothelial cells showed that ZnO nanoparticles (20–70 nm diameter) can be internalized into cells and that ZnO-induced inflammatory response is due to the presence of the particles rather than ZnO-released Zn^2+^ (Gojova et al. 2007). In contrast, a recent study using Raw264.7 cells and BEAS-2B cells proposed that the toxicity of ZnO nanoparticles (13 nm) in both cell types is related to particle dissolution that could happen in culture medium and intracellular endosomes (Xia et al. 2008). In separate experiments (data not shown) we observed that ZnO particles were poorly dissolved in water and that the phagocytosis inhibitor cytochalasin D partially blocked ZnO-induced *IL-8* expression in BEAS-2B cells, implying that ZnO particle internalization and subsequent dissolution may be involved in ZnO-induced *IL-8* expression.

Human inhalation studies have shown that exposure to ZnO in welding fumes induced early increase in TNFα protein concentration and subsequent elevation of IL-8 and IL-6 protein levels in bronchoalveolar lavage fluid (Blanc et al. 1993; Kuschner et al. 1997). Moreover, TNFα has been shown to induce IL-8 production from BEAS-2B cells (Fujisawa et al. 2000). These observations lead to an assumption that ZnO-induced *IL-8* expression may be mediated through an autocrine mechanism that involves TNFα. However, we have observed that TNFα-neutralizing antibody had minimal inhibitory effect on ZnO-induced *IL-8* mRNA and protein expression even though it blocked TNFα-induced *IL-8* mRNA expression by > 90% (data not shown), implying that TNFα is not involved in ZnO-induced *IL-8* expression in BEAS-2B cells.

ZnO usually exists in the form of ultrafine particles in ambient and workplace air; thus, exposure to ZnO particles is associated with adverse effects in environmental and occupational settings. Characterization of the underlying mechanisms of ZnO toxicity may provide helpful information in the prevention and treatment of pulmonary and systemic disorders related to inhalation of ultrafine ZnO particles.
